# Loss of Saltation and Presynaptic Action Potential Failure in Demyelinated Axons

**DOI:** 10.3389/fncel.2017.00045

**Published:** 2017-02-27

**Authors:** Mustafa S. Hamada, Marko A. Popovic, Maarten H. P. Kole

**Affiliations:** ^1^Department of Axonal Signaling, Netherlands Institute for Neuroscience, Royal Netherlands Academy of Arts and SciencesAmsterdam, Netherlands; ^2^Cell Biology, Faculty of Science, Utrecht UniversityUtrecht, Netherlands

**Keywords:** axon, axon collaterals, boutons, node of Ranvier, action potential, demyelination

## Abstract

In cortical pyramidal neurons the presynaptic terminals controlling transmitter release are located along unmyelinated axon collaterals, far from the original action potential (AP) initiation site, the axon initial segment (AIS). Once initiated, APs will need to reliably propagate over long distances and regions of geometrical inhomogeneity like branch points (BPs) to rapidly depolarize the presynaptic terminals and confer temporally precise synaptic transmission. While axon pathologies such as demyelinating diseases are well established to impede the fidelity of AP propagation along internodes, to which extent myelin loss affects propagation along BPs and axon collaterals is not well understood. Here, using the cuprizone demyelination model, we performed optical voltage-sensitive dye (VSD) imaging from control and demyelinated layer 5 pyramidal neuron axons. In the main axon, we find that myelin loss switches the modality of AP propagation from rapid saltation towards a slow continuous wave. The duration of single AP waveforms at BPs or nodes was, however, only slightly briefer. In contrast, by using two-photon microscopy-guided loose-seal patch recordings from axon collaterals we revealed a presynaptic AP broadening in combination with a reduced velocity and frequency-dependent failure. Finally, internodal myelin loss was also associated with *de novo* sprouting of axon collaterals starting from the primary (demyelinated) axon. Thus, the loss of oligodendrocytes and myelin sheaths bears functional consequences beyond the main axon, impeding the temporal fidelity of presynaptic APs and affecting the functional and structural organization of synaptic connectivity within the neocortex.

## Introduction

Action potentials (APs) are the primary signals by which neural information is electrically encoded and distributed throughout the nervous system to provide temporally precise neurotransmitter release at the presynaptic terminals. In most neurons, the presynaptic terminals are anatomically dispersed along the branches of axon collaterals, far away from the original AP initiation site in the axon initial segment (AIS; Kole and Stuart, 2012). Such anatomical organization requires the AP voltage transient to reliably propagate when covering long distances from the AIS until the transmitter release sites. Such reliability is particularly important considering that neural coding strategies in the neocortex are often relying on a sparse coding scheme, in which low mean firing rates of individual neurons represent features of sensory modalities (Brecht et al., 2004; Harris and Mrsic-Flogel, 2013), the temporal fidelity of how APs propagate from their initiation site into complex branched axonal trees and the presynaptic terminal limits the capacity for neural information encoding.

AP propagation is well known to fail at low-safety conduction points including branch points (BPs), regions of inhomogeneity or abrupt diameter changes in an activity-dependent manner (Goldstein and Rall, 1974; Parnas and Segev, 1979; Deschênes and Landry, 1980; Manor et al., 1991; Ducreux et al., 1993; Debanne et al., 1997). These observations led to the general notion that the temporal features of neuronal firing in highly branched axon terminals may differ from their main axon (Deschênes and Landry, 1980). AP propagation fidelity is furthermore strongly compromised in axon pathologies such as demyelinating diseases. In the spinal cord, optic nerve, corpus callosum and neocortex myelin loss causes a substantial slowing in conduction velocity (McDonald and Sears, 1970; Bostock and Sears, 1978; Felts et al., 1997; Crawford et al., 2009; Hamada and Kole, 2015). However, whether AP propagation into the higher-order small-diameter collaterals, where all synaptic terminals reside, is equally impaired remains poorly understood. Axon collaterals are typically unmyelinated but given their complex branched geometries, we hypothesized that they may be particularly sensitive to demyelination-induced failures in high-frequency firing (Bostock and Sears, 1978; Kim et al., 2013). In addition, demyelination produces a large variability in both nodal anchoring proteins (Ankyrin G and βIV-spectrin) and their voltage-gated ion channel expression (Nav and Kv7) along individual axons (Hamada and Kole, 2015), possibly impairing invasion of collaterals. Transmission failures at BPs could also arise from local Nav channel inactivation causing frequency-dependent failure during high-frequency firing in axons (Monsivais et al., 2005; Khaliq and Raman, 2006).

In order to determine the impact of myelin loss on AP fidelity in the main axon and their collateral branches, we performed optical voltage-sensitive dye (VSD) imaging and two-photon microscopy-guided patch-clamp recordings from en passant presynaptic boutons in layer 5 axon collaterals. We find that myelin loss causes a frequency-dependent failure of APs at the presynaptic terminals. In addition, axon collaterals in demyelinated axons showed *de novo* sprouting. Taken together, demyelination affects intra-cortical synaptic transmission beyond a slowing of AP signaling in the originally myelinated main axon.

## Materials and Methods

### Electrophysiological Recordings and Two-Photon Imaging

Male C57BL/6 mice were kept on a 12:12 h light-dark cycle and brain slices prepared at ~3 h after onset of the light period. A total of 20 mice were used (control, *n* = 9, 11 weeks; cuprizone, *n* = 11, 11 weeks). Mice in the cuprizone experimental group were fed with 0.2% cuprizone for 5 weeks supplemented in the diet and acute slice preparation and recording from layer 5 pyramidal neurons in somatosensory cortex were performed in cortical slices (300 μm) as previously described (Hamada and Kole, 2015). All experiments and protocols were in compliance with the European Communities Council Directive of 24 November 1986 (86/609/EEC) and were reviewed and approved by the animal welfare and ethics committee (DEC) of the Royal Netherlands Academy of Arts and Sciences (KNAW) under the protocol number NIN 11.70.

Two-photon (2P) visualization and electrophysiological recordings were performed using galvanometer-based laser-scanning microscope (Femto3D-RC, Femtonics Inc., Budapest, Hungary). A Ti:Sapphire pulsed laser (Chameleon Ultra II, Coherent Inc., Santa Clara, CA, USA) tuned to 800 nm was used for two-photon excitation. Three photomultipliers (PMTs, Hamamatsu Photonics Co., Hamamatsu, Japan) were used for signal detection, two were used to collect the fluorescence signals and one to scan the transmitted IR-light (800 nm). The two signals were overlaid for fluorescence-assisted patching from axons. Whole-cell somatic patch-clamp pipettes were filled with intracellular solution containing 200 μM Alexa Fluor 568 hydrazide (Sigma-Aldrich) to visualize cellular morphology. Layer 5 pyramidal neurons in the primary somato-sensory cortex were filled for at least 30 min in whole-cell configuration before commencing with dual soma-axon recording approaches. For axonal loose-seal patch-clamp recordings the signals were filtered at 2 kHz using a Multiclamp 700B (Molecular Devices Co., Sunnyvale, CA, USA). Loose-seal recordings from visually identified BPs and *en passant* boutons were accomplished by gently pressing the pipette tip (~10 MΩ open tip resistance) against the membrane and applying negative pressure to form a seal resistance between ~30 and 50 MΩ. All recordings were made at 32 ± 1°C. Electrophysiological data were digitized (ITC-18 InstruTECH, HEKA Elektronik GmbH) at 100 kHz and acquired using AxoGraph X (v. 1.5.4, Molecular Devices Co., Sunnyvale, CA, USA). APs were analyzed using custom-written routines in MatLab (The MathWorks Inc., Natick, MA, USA). High frequency somatic APs were evoked by brief (1 ms) square currents pulses with decreasing inter-pulse intervals. To convert loose-seal recorded APs into binary probability values, the failure of propagation was determined using a semi-automatic routine with a threshold set at ~25% of the maximal amplitude of the first evoked bouton AP, confirmed by visual inspection of each individual spike.

### Immunohistochemistry and Confocal Microscopy

Live imaging of primary axons of layer 5 pyramidal neurons was done using 2P laser-scanning microscope (Femto3D-RC, Femtonics Inc., Budapest, Hungary). Neurons were loaded with dye (200 μM Alexa 568) for at least 30 min to allow sufficient diffusion into the primary axon and collaterals. For morphological analysis, a z-stack with a 300 × 300 μm field of view was scanned at a resolution of 1000 × 1000 pixels (1 μm z-step) using a 60× (NA1.0) water-immersion objective. Images were saved as TIFF files and later processed using Fiji (ImageJ) graphics software (v1.47p, NIH). Immediately after recording and imaging slices were transferred to a fixative containing 4% paraformaldehyde (20 min). Brain slices were then blocked with 10% bovine serum albumin (BSA), 5% normal goat serum (NGS) and 2% Triton X-100 for 2 h at room temperature (RT) before incubation with mouse anti-myelin basic protein (MBP; 1:250; Covance Inc., Princeton, NJ, USA), rabbit anti-βIV-spectrin (1:250, gift from M. N. Rasband, Baylor College of Medicine, Houston, TX, USA), fluorophore-conjugated streptavidin (1:500; Invitrogen B.V., Groningen, Netherlands) for 24 h, followed by second antibody incubation for 2 h (488 goat anti-rabbit, streptavidin-conjugated 568, and 633 goat anti-mouse, Invitrogen B.V., Groningen, Netherlands). Confocal images of labeled neurons were collected with a Leica TCS SP8 X confocal laser-scanning microscope (Leica Microsystems GmbH) at 2048 × 2048 pixels (0.5 μm z-step) using a 40× (1.3 N.A., 0.75–1.0 digital zoom) oil-immersion objective. To avoid bleed-through between emission wavelengths, automated sequential acquisition of multiple channels was used.

### Optical Voltage-Sensitive Dye Recordings

Individual layer 5 pyramidal neurons from somatosensory cortex were selectively labeled with a membrane impermeable VSD by allowing free diffusion of the probe from a somatic patch pipette in the whole-cell configuration. We used the most successful voltage probe for intracellular application, JPW3028, which is a doubly positively charged analog of the ANEP series of lipophilic styryl dyes that is still sufficiently water soluble to be used for microinjection. Its close analog JPW1114 characterized by the same voltage sensitivity is commercially available (Catalog number D6923, Invitrogen, ThermoFisher Scientific Inc., Waltham, MA, USA). Patch pipettes were first filled with the dye-free solution to about three quarters of the pipette taper and then filled with the solution containing the indicator dye (0.8 mM). Intracellular filling was accomplished in 30–60 min. To stimulate and obtain electrical recordings from the soma, the cell body was re-patched using an electrode filled with dye-free intracellular solution before making optical measurements. We used a stationary upright microscope (Olympus BX51WI, Olympus, Japan) equipped with two camera ports. One camera port had a high spatial resolution CCD camera for oblique contrast video-microscopy (CoolSNAP EZ, Photometrics). The second camera port had a fast data acquisition camera with relatively low spatial resolution (80 × 80 pixels) but outstanding dynamic range (14 bits) and exceptionally low read noise (NeuroCCD-SM, RedShirtImaging LLC, Decatur, GA, USA).

The brain slice was placed on the stage of the microscope and the fluorescent image of the stained neuron projected by a water immersion objective (100×/1.1 NA, Nikon, Japan) onto the fast data acquisition CCD positioned in the primary image plane. This objective was selected as a compromise between imaging area, spatial resolution, and signal-to-noise ratio (S/N). Optical recording of VSD signals from the axonal arbor was carried out in the wide-field epifluorescence microscopy mode. A frequency-doubled 500 mW diode-pumped Nd:YVO4 continuous wave laser emitting at 532 nm (MLL532, Changchun New Industries Optoelectronics Tech. Co., Ltd., Changchun, China) was the source of excitation light. The laser beam was directed to a light guide coupled to the microscope via a single-port epifluorescence condenser designed to provide approximately uniform illumination of the object plane (assembly adapted from X-Cite^®^ 120Q lamp, Excelitas Technologies Co., Waltham, MA, USA). The fractional noise of low-noise solid-state lasers (RMS < 0.5%) is below typical fractional shot-noise in fluorescence VSD recordings. The laser was used as a light source in place of a conventional Xenon arc-lamp to maximize the sensitivity of optical *V*_m_ imaging by: (1) using a monochromatic excitation light at the red wing of the absorption spectrum to maximize *V*_m_ sensitivity of the dye; and (2) increasing the intensity of the excitation light beyond the level that can be achieved by an arc-lamp. The excitation light was reflected to the preparation by a dichroic mirror with the central wavelength of 560 nm, and the fluorescence light was passed through a 610 nm barrier filter (parts of Olympus U-MWG filter assembly cube). The image of a stained neuron was projected onto a CCD chip via a 2× intermediate zoom (U-ECA, Olympus) and 0.1× de-magnifier (RedShirtImaging LLC, Decatur, GA, USA). The CCD frame (26 × 4 pixels) corresponded to approximately an 82.5 × 12.7 μm area in the object plane with each individual pixel receiving light from an area of ~3.2 × 3.2 μm. To measure the AP shape in the nodes of Ranvier (noR) changes in the light intensity were recorded while an AP was evoked by brief (3 ms) transmembrane current pulse with the intensity tuned to trigger an AP in the cell delivered via a recording electrode attached to the soma in whole-cell current-clamp configuration. Signal averaging (~80–100 trials) was used to improve the S/N further.

### Statistical Analysis

Data are given as mean ± SEM, and were statistically analyzed with MatLab 2014a (The MathWorks, Natick, MA, USA) or IGOR Pro 6, WaveMetrics). When *n* ≥ 6, data were tested for normal distribution with a Shapiro-Wilk test and when positive, we applied an unpaired *t*-test subsequently. Not normally distributed experiments with *n* < 6 were tested with non-parametric unpaired Mann-Whitney (M-W) test. Cut-off significance level (*P*) was set to 0.05. Pearson correlation coefficients were determined using IBM SPSS (v.23; IBM Co., New York, NY, USA).

## Results

### Non-Saltatory Reliable Propagation of APs in the Demyelinated Main Axon

**Figure 1:**
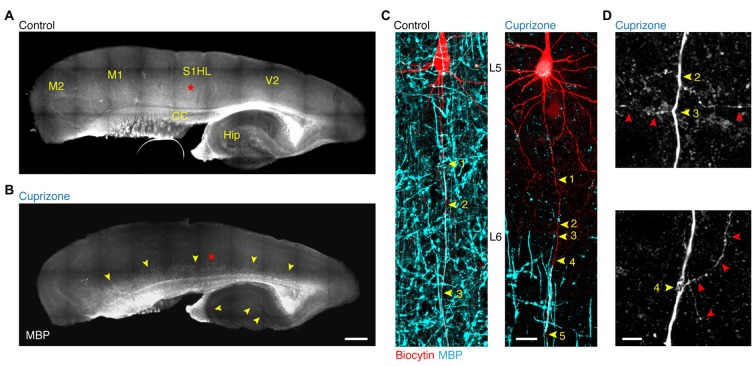
**Demyelination of the main axon of thick-tufted layer 5 pyramidal neurons. (A,B)** Overview fluorescent image of a myelinated **(A)** demyelinated **(B)** parasagittal brain section immunolabeled for myelin basic protein (MBP). *Red asterisks*, indicate the locations of the recorded thick-tufted layer 5 neuron shown in **(C)**. Note that cuprizone-induced gray matter demyelination (*yellow arrowheads*) occurs across the entire cerebral cortex. S1HL, primary somatosensory hindlimb cortex; V2, secondary visual cortex; M1, primary motor cortex; M2, secondary motor cortex; CC, corpus collosum; Hip, hippocampus. Scale bar, 700 μm, **(C)** *z*-projected confocal images of layer 5 axons co-labeled for biocytin (*red*) and MBP expression (*cyan*). *Yellow arrowheads*, indicate branch points (BPs) along the main axon. Scale bar, 20 μm. **(D)** Magnified *z*-projected confocal images of BPs of the demyelinated primary axon shown in **(C)**. *Yellow arrowheads*, BPs. *Red arrowheads*, trajectory of the primary and secondary axon collaterals. Scale bar, 5 μm.

In order to study the forward propagation of the AP in single layer 5 axons, we made targeted recordings from visually identified layer 5 neurons in the primary somatosensory hindlimb region (S1HL) of demyelinated brain slices (Figures 1A,B). Widefield imaging of parasagittal slices including neocortex and hippocampus revealed that 5 weeks of 0.2% cuprizone treatment causes widespread loss of MBP, in particular in lamina 2/3 and 5 of the neocortex as well as in the hippocampus (Clarner et al., 2012; Dutta et al., 2013; Hamada and Kole, 2015). Confocal *z*-projected images of electrophysiologically recorded and biocytin-filled thick-tufted layer 5 neurons shows the extent of myelin loss at internodes of cuprizone-treated animals (Figure 1C). At the single axon resolution the first 3–5 internodes were rarely covered by myelin, consistent with previous work (Hamada and Kole, 2015). Therefore, the proximal region of the main layer 5 pyramidal neuron axon provides a reliable and reproducible domain to explore mechanisms and consequences of myelin loss in internodes and the associated axon collaterals (Figure 1D).

**Figure 2:**
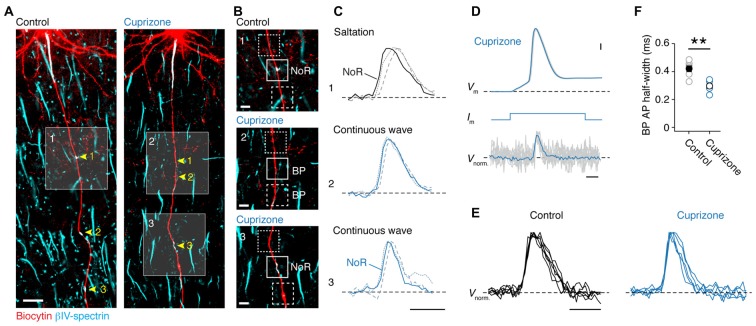
**Loss of saltatory propagation and narrowing of nodal action potentials (APs) in demyelinated axons. (A)** *Z*-projected confocal images of primary layer 5 axons immuno-labeled for (biocytin, *red*) and βIV-spectrin (*cyan*). Nodes of Ranvier (noR) in myelinated (*left*) and BPs in demyelinated (right) axons are highlighted. Scale bar, 20 μm. **(B)** Zoomed in regions of interest indicated by white squares in **(A)**. Examples of normal noR pattern in control mice (*top*) and BPs from cuprizone treated mice: lacking βIV-spectrin (*middle*) and βIV-spectrin enriched (*bottom*). Scale bar, 5 μm. **(C)** *Top*: normalized voltage-sensitive dye (VSD) traces from noR (*solid line*), preceding internode (*dotted line*) and following internode (*dashed line*). *Middle* and *bottom*: normalized VSD traces from BP (*solid line*), more proximal area (*dotted line*) and more distal area (*dashed line*). Scale bar, 0.5 ms. **(D)** Align and overlay of nine example (out of 100) somatic APs and their corresponding BP spikes (VSD recorded at 20 kHz, gray) and the average of all 100 recorded traces (*black*). Somatic single APs (*top*) were elicited through brief (3 ms; *middle*) square current pulse. BP, branch point. Scale bar, 0.5 ms; 10 mV. **(E)** Normalized VSD traces from control (5 noR; *n* = 5 cells) and cuprizone-treated mice (6 BPs; *n* = 5 cells). Scale bar, 0.5 ms. **(F)** Comparison of BP AP half-widths in control and cuprizone treated mice obtained from VSD data. APs in cuprizone treated mice are significantly narrower (control, *n* = 5 noR; cuprizone, *n* = 6 BPs; Mann-Whitney (M-W) test, **P = 0.0087). Individual cells plotted as open circles.

Although direct whole-cell patch-clamp recordings from axon blebs (Kole et al., 2007; Kole and Popovic, 2016) provides the best possible temporal resolution to examine axonal APs, mouse axons are small in diameter (<1.0 μm) making this approach technically challenging. In addition, demyelination is accompanied by a significant diminution of axon diameter (Mason et al., 2001), as well as aberrant axon neurofilament (de-) phosphorylation (de Waegh et al., 1992; Smith et al., 2013), which may explain the lack of swellings at the cut ends of axons. As an alternative, we here used the approach of optical VSD imaging (Popovic et al., 2011). We obtained reliable optical signals of *V*_m_ in the main axon and correlated signals with the expression of anti-βIV-spectrin in these axons (Figure 2A). Optical signals of *V*_m_ were observed in internodes and noR from control axons (*n* = 5 cells; Figures 2B,C, top), in βIV-spectrin-negative BPs in demyelinated axons (*n* = 3 cells; Figures 2B,C, middle) as well as in βIV-spectrin-enriched BPs (*n* = 2 cells; Figures 2B,C, bottom). To improve the S/N ratio, up to 100 trials were averaged for individual recordings. In all control axons saltatory AP conduction could be observed as *V*_m_ in the node always temporally preceded the previous internodal AP signal (Figure 2C, top). These findings confirm previous observations of saltation in adult myelinated layer 5 axons in mice (Popovic et al., 2011). In contrast, spatiotemporal analysis of the AP waveforms in axons from cuprizone-treated animals revealed a continuously propagating wave compared to the preceding and succeeding internodes (Figure 2C, middle and bottom). Continuous propagation occurred independent of the presence or absence of βIV-spectrin expression suggesting that only the myelin sheath is essential for temporal saltation. Interestingly, *post hoc* immunolabeling of one axon, which did show saltatory conduction, revealed partial internodal (re-) myelination (data not shown). Taken together, these results suggest that the presence of myelin is crucial for saltatory conduction.

In addition to probing the spatial properties of the AP propagation, optical *V*_m_ recording with the voltage probe JPW3028 also enables reliable assessment of the intracellular AP shape at ~20 μs resolution (Popovic et al., 2011). As every BP in demyelinated axons showed APs, we compared the half-widths of the optical *V*_m_ signals from putative nodal BPs (Figures 2D,E). Interestingly, the AP half width was significantly ~100 μs narrower in the BPs from demyelinated axons (Figure 2F). Taken together, these results indicate reliable, albeit slow, propagation of APs in demyelinated main axons, which are slightly shorter in half-width duration.

### Single APs Successfully Invade the Axon Collaterals of Demyelinated Axons

**Figure 3:**
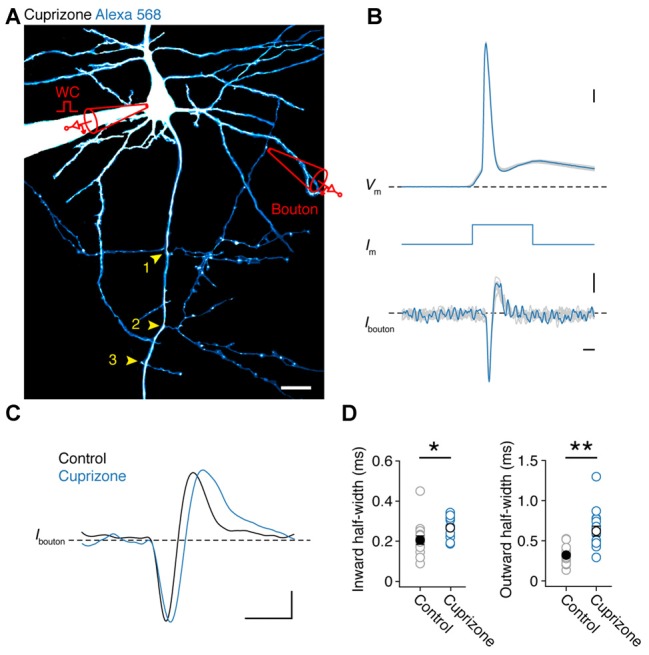
**Presynaptic bouton recordings reveal AP broadening in axon collaterals. (A)** 2P fluorescence overview image of a demyelinated layer 5 pyramidal neuron loaded with Alexa 568 (200 μM), *Yellow arrows*, BP locations. Image has been modified for clarity by subtracting the background noise extensively to highlight the collateral tree. A loose-seal recording is indicated schematically. Scale bar, 20 μm. **(B)** Temporally aligned overlay of eight somatic APs and their corresponding bouton axonal spikes. Bouton loose-seal patch APs (*bottom*) were recorded by repetitively eliciting somatic single APs (*top*) through brief (3 ms; *middle*) square current pulse. Scale bar, 0.5 ms; 10 mV; 5 pA. **(C)** Example traces of aligned APs recorded in control (*black*) and demyelinated (*blue*) axon collaterals (~149 μm from the AP initiation). Scale bar, 1 ms; 5 pA. **(D)** Plots of the average half-widths of the inward and outward current components of the recorded bouton APs. Cuprizone, *blue circles*, *n* = 16 boutons from 14 cells. Control, *gray circles*, *n* = 15 boutons from 14 cells. Individual recordings plotted as open circles. M-W test, *P = 0.0010, **P = 0.0002, respectively. Data are presented as mean ± SEM.

If APs are detectable in all nodes of demyelinated axons, independent of spectrin expression, this suggests they will reliable invade collaterals. As JPW3028 diffusion into the thin axon collaterals is limited (but see Rowan et al., 2016) we made simultaneous somatic and two-photon (2P)-targeted loose-seal patch recordings from en passant presynaptic boutons (Figure 3A). After approximately 30 min of somatic whole-cell recording with a fluorescent dye (200 μM Alexa 568), BPs of layer 5 axons, which commonly arise at noR (Sloper and Powell, 1979; Fraher and Kaar, 1984), could be imaged with 2P laser scanning microscopy and collaterals with visually detectable boutons targeted for loose-seal voltage-clamp recordings under continuous 2P imaging at distances between 90–580 μm from the AP initiation site (26 μm from the soma (Hamada and Kole, 2015); *n* = 31 boutons from 30 cells). Somatically elicited APs were visible in the boutons as rapid capacitive currents corresponding to the depolarizing and repolarizing phase of the underlying AP. An overlay of all somatic and bouton APs shows that each event in the collateral was associated with an AP triggered at the soma, confirming a near 100% reliability in propagation (31 out of 31 boutons; Figures 3A,B). While the local APs waveforms at boutons from control and demyelinated axons were not different in amplitude (Inward current amplitude: cuprizone, −18.8 ± 4.0 pA, *n* = 16; control, −12.1 ± 1.4 pA, *n* = 15; *P* = 0.1160; outward current amplitude: cuprizone, 10.7 ± 1.0 pA; control, 8.0 ± 1.3 pA; *P* = 0.3726) they showed a significantly longer half-width duration in both the inward and outward component compared to control (Figures 3C,D; *P* = 0.010 and *P* = 0.0002, respectively). These data suggest that presynaptic APs in the unmyelinated collaterals are broader and thereby oppositely affected when compared to the nodal APs in the main axon.

**Figure 4:**
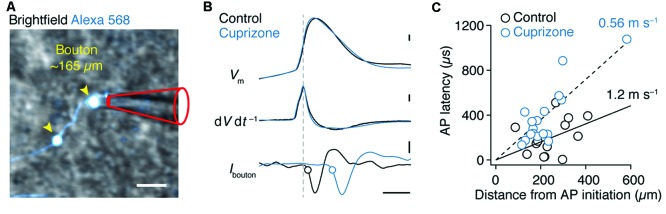
**Reduced conduction velocity in axon collaterals of demyelinated layer 5 axons. (A)** Magnified 2P scan overlaid with the brightfield image from a demyelinated layer 5 axon a distance of ~165 μm from the soma. Scale bar, 2 μm. **(B)** Top, somatically evoked single APs from control and demyelinated layer 5 neurons. Middle, time derivative of the somatic APs aligned at peak amplitude. Bottom, loose-seal patch recording of bouton APs recorded at ~300 μm from the soma of respective neurons. Note the delay of the AP in demyelinated axon due to reduced conduction velocity as a consequence of myelin loss. *Closed circles* indicate the 20% onset of the local spike maxima. Scale bar, 0.5 ms; 10 mV; 1 kV s^−1^; 5 pA. **(C)** Axosomatic latency plotted vs. total measured bouton distance (measured from the AP initiation site, 26 μm). Control (*n* = 13 boutons from 13 cells; *open circles*) and cuprizone data sets (*n* = 17 boutons from 15 cells; *blue open circles*) are fitted with a linear function.

We next investigated the AP conduction velocity in axon collaterals. Average spike-triggered current transients recorded near each en passant bouton were aligned to the peak d*V* d*t*^−1^ of the somatic AP. Axo-somatic conduction delays were calculated relative to the 20% rise point of each averaged bouton spike (Hamada and Kole, 2015) and plotted vs. the distance from the AP initiation site in micrometers measured in 3D image stacks (Figures 4A,B). The AP latencies were fitted with a linear function yielding an estimate of axon-collateral conduction velocity of 0.56 ms^−1^ in demyelinated axons, a two-fold velocity reduction compared with the control velocity of 1.2 ms^−1^ (Figure 4C).

### Increased AP Failure in Demyelinated Axon Collaterals during High-Frequency Stimulation

**Figure 5:**
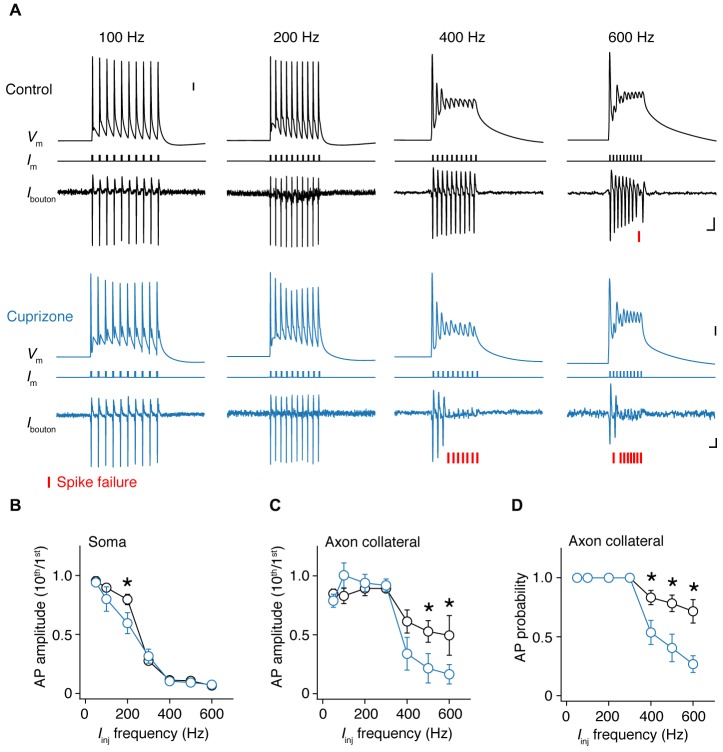
**Frequency-dependent AP failures in presynaptic boutons of demyelinated axons. (A)** Simultaneous somatic whole-cell and axonal loose-seal recording from control (*black*) and demyelinated neurons (*blue*) during somatic current injections (1 ms pulses) at increasing frequencies. Note the increased failure rate in demyelinated axons. *Asterisks*, spike failure. Scale bar, 5 ms; 10 mV; 10 pA. Somatic capacitive transients are blanked for clarity reasons. **(B)** Normalized somatic AP amplitude vs. injected current frequency. *t*-test, *P = 0.0432. **(C)** Collateral AP amplitude vs. evoked injected current frequency. M-W test, *P = 0.0446 (500 Hz); *P = 0.0426 (600 Hz). **(D)** Relationship between AP probability vs. somatic step frequency in control (*n* = 16 boutons from 16 cells; *black open circles*) and demyelinated axons (*n* = 14 boutons from 14 cells;* black open circles*). Data presented as average ± SEM. M-W test, *P* = 0.0185 (400 Hz); *P* = 0.0098 (500 Hz); *P* = 0.0063 (600 Hz).

As shown in Figure 3B, single APs propagate into the axon collateral with 100% fidelity. However, at higher frequencies we noticed that spikes within a train started to show failures (Figure 5A). To probe this relationship between frequency and failures quantitatively, we elicited APs by injecting 10 square current steps in the soma (amplitude range: 4–6 nA; duration: 1 ms), with increasing frequencies (50, 100 to 600 Hz, steps of 100 Hz) and repeated these ~40 times to average the traces and increase the S/N ratio. To quantify the frequency at which failures occur, we examined whether the somatic compartment can faithfully track APs during high-frequency current injection (Figure 5B). By comparing the amplitude of the last AP (10th) to the first initiated AP (1st), we found a sharp drop in the somatic AP amplitude at 300 Hz, and at higher frequencies the soma failed completely to initiate APs in both groups. However, control neurons displayed less amplitude attenuation at 200 Hz at the soma when compared to demyelinated neurons (Figure 5B).

Recordings at the axon showed that AP amplitude attenuation was less prominent at higher input frequencies (400–600 Hz) when compared to the soma consistent with the possibility of axonal Nav channels to recover more quickly from Nav channel inactivation and the depolarized voltage threshold for activation of the somatodendritic spike component (Kole and Stuart, 2008; Popovic et al., 2011; Figures 5A,C). However, in this frequency range, presynaptic AP recordings from demyelinated axons showed significantly larger amplitude attenuation when compared to control axons (Figure 5C). Finally, we examined the inward amplitude component of the axonal signals, converted the analog axonal signals into binary data and plotted AP probability against the input frequency (Figure 5D; see *Experimental Procedures*). The results showed that presynaptic AP recordings from demyelinated axons. The axonal AP failures occurred in the same range of input frequencies but for significantly more APs in the train (at 600 Hz, cuprizone ~70% failure vs. control 30%; *P* = 0.0063; Figure 5D). Furthermore, axonal AP failures at any given input frequency did not correlate with recording distance from the soma (Pearson correlation; control: *r* = −0.1669, *P* = 0.6449 (500 Hz); cuprizone, *r =* −0.2585, *P* = 0.5365 (500 Hz)).

### Structural Plasticity of Axon Collaterals

**Figure 6:**
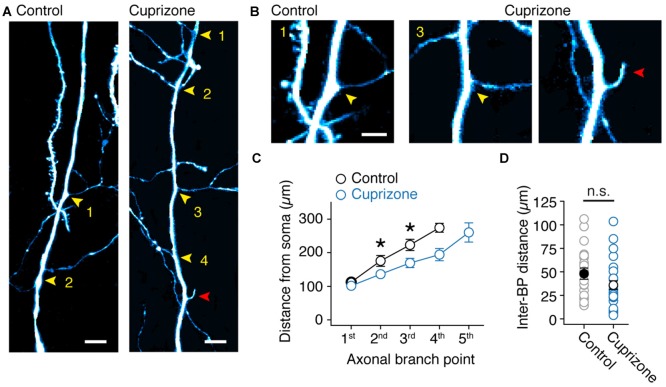
**Sprouting of new axon collaterals in demyelinated axons. (A)** Fluorescent 2P images of the proximal BPs in control and demyelinated layer 5 axons. Note the larger number of BPs in demyelinated axon compared to control. *Yellow arrowheads*, BPs. *Red arrowhead*, putative *de novo* axon outgrowth. Scale bar, 10 μm. **(B)** Magnified 2P images of the same images shown in **(A)**. *Yellow arrowheads*, BPs; *Red arrowhead*, putative *de novo* axon outgrowth. Scale bar, 5 μm. **(C)** Plot of the BP locations within the first 350 μm of primary axon (measured from the soma). M-W test, *P = 0.0301 (control, *n* = 11 BPs; cuprizone, *n* = 13 BPs; 2nd branch point); *P = 0.0079 (control, *n* = 10 BPs; cuprizone, *n* = 11 BPs; 3rd branch point). Data presented as mean ± SEM. **(D)** Plot of the average inter-BP distance. Control, *black open circles*, *n* = 24 inter-BP distances from 11 cells. Cuprizone, *blue open circles*, *n* = 28 inter-BP distances from 12 cells. M-W test *P* = 0.1670.

Based on the axon morphologies acquired during 2P imaging we noticed that BPs in demyelinated axons appeared denser. To test the hypothesis that myelin loss affects the anatomical organization of the main (de-)myelinated axon arbor we examined the BP locations within the first 300 μm from the soma using high-resolution two-photon imaging (Figure 6A). Similar to a previous anatomical study of thick-tufted layer 5 axons in the adult rat (Romand et al., 2011), we found that the main axon has approximately three BPs from which horizontal collaterals emerge. On average, the total number of BPs were not different between control and demyelinated axons (M-W test, *P* = 0.3715; control, 3.2 ± 0.2 BPs, *n* = 11 axons; cuprizone, 3.6 ± 0.3 BPs, *n* = 14 axons; Figure 6A, yellow arrowheads). Interestingly, a few demyelinated axons showed a large number of BPs and were characterized by protrusions indicative of axonal sprouting (*n* = 2 sprouting axons, Figure 6B; red arrowhead). When plotting the BP locations as a function of distance from the soma demyelinated axons showed a significant shift towards a more proximal onset for both the second and third BP (*P* = 0.0079, *P* = 0.030, respectively; Figure 6C). Furthermore, while the average inter-BP distance was not different between the two groups (control, 48.1 ± 6.0 μm; cuprizone, 36.0 ± 4.6 μm; *P* = 0.1670; Figure 6D), the first inter-BP distance was on average ~30 μm shorter in demyelinated axons (control, 57.0 ± 9.0 μm; cuprizone, 29.0 ± 4.8 μm;* t*-test, *P* = 0.0095). This was not different anymore for the second inter-BP length (control, 40.8 ± 7.9 μm; cuprizone, 31.8 ± 7.3 μm; *t*-test, *P* = 0.4112). These data indicate that collaterals and internodes around the first BPs from the soma undergo structural plasticity in demyelinated axons.

## Discussion

In this study we addressed how APs are propagating along demyelinated internodes and variably reorganized nodal domains to reach the presynaptic terminals. Axon-glia interactions at the contact sites between the axon and myelin sheath are critical for the proper assembly and maintenance of the microdomains of the node of Ranvier (Poliak and Peles, 2003; Chang et al., 2014). As a consequence of the loss of the myelin sheath in experimental demyelination models or in MS, nodal regions are characterized by a large redistribution or *de novo* expression of anchoring proteins and voltage-gated ion channels (Rasband et al., 1998; Arroyo et al., 2002; Craner et al., 2004; Black et al., 2006; Hamada and Kole, 2015). Interestingly, along the successive BPs of a single axon a large diversity in the expression of ion channel proteins can be found from node to node (Hamada and Kole, 2015). Here, we found that molecular variation in nodes did, however, not greatly impact on conduction along the main axon; independent of βIV-spectrin myelin loss was found to switch the optically recorded AP from rapid saltation into a continuous non-saltatory wave (Figure 2). Continuation of AP propagation in demyelinated main axons is consistent with the observation that even low Nav channel densities at the denuded internode axolemma suffice to ensure propagation of axonal APs (Shrager, 1993).

The biophysical basis underlying the ~120 μs narrower half-width at demyelinated BPs most likely relies on redistribution of juxtaparanodal Kv channels (Rasband et al., 1998; Arroyo et al., 2002; Black et al., 2006; Bagchi et al., 2014). However, technical errors in measuring voltage accurately at these small domains may also play a confounding factor. First, the local amplitude of optically recorded APs is unknown because the fractional fluorescence change depends on the surface to volume ratio of imaged compartments. Secondly, the pixel size encompassing a region of 3.2 μm^2^, is slightly larger compared to the ~2.5 μm^2^ size of the noR in control axons (Hamada and Kole, 2015) and thus may contain fluorescence emitted from the internode either under the myelin sheath or, in the case of cuprizone, the exposed internodal axolemma. Decreasing the pixel size would, however, either reduce the temporal resolution, causing undersampling and aggravate the averaging jitter, or require further zooming which would reduce the field of view to <50 μm, decrease the fluorescence light intensity and, hence, the S/N ratio. To compensate for such light loss, tissue-damaging levels of excitation light would be required. Taken together, the current approach was the best available compromise between pixel size, temporal resolution and S/N ratio.

The most likely explanation for the optically recorded narrower AP is the increased expression and redistribution of fast-activating Kv1.1/Kv1.2 channels in demyelinated axons (Rasband et al., 1998; Arroyo et al., 2002; Black et al., 2006; Bagchi et al., 2014). This hypothesis remains to be tested by comparing recordings with and without Kv1 channel blockers. Kv1 channels are typically clustered at the juxta-paranodal domains, but with myelin loss disperse into the paranodes and nodal axolemma or in opposite direction into the internode (Rasband et al., 1999; Rasband and Shrager, 2000). Furthermore, in the demyelinated optic nerve in cuprizone mouse model, Kv1.1 homo-tetramers undergo *de novo* expression in denuded axons (Bagchi et al., 2014). Additionally, the same study also showed that Kv1.1 subunit homo-tetramers confer a decrease in the activation voltage threshold and accelerate the activation kinetics. Since axonal Kv1 voltage-gated potassium channels play a key role in repolarizing the axonal AP (Kole et al., 2007; Foust et al., 2011), their expression in the nodal axolemma may significantly shorten the AP half-width duration and rapid repolarization would act to increase the Nav channel availability during repetitive firing.

In striking contrast to the AP waveform in the main axon recordings from the presynaptic boutons revealed a broadening of the presynaptic AP current components (Figure 3). The molecular changes underlying presynaptic AP broadening are not clear but, similar to the main axon, may depend on expression changes in Kv1- or Kv3-subtype potassium channels. Staining and/or recording Kv1/3 channels in presynaptic terminals is challenging and there is no information about their expression in demyelination models. Recently, it was shown that bouton-specific expression of fast-activating Kv3 channels causes large heterogeneity of presynaptic AP durations along successive boutons of axon collaterals (Rowan et al., 2014, 2016). In addition to the broadening of presynaptic APs at boutons from demyelinated axons the conduction velocity was also significantly lower, indicating that myelin loss at the main axon affects arrival times into the axon collaterals impeding on the temporal precision of glutamatergic excitation of the target cells (Figure 4).

While at frequencies near 300 Hz AP failure at the soma rapidly increases with successive spikes, neocortical axons can generate higher firing rates (Figure 5; Popovic et al., 2011). Interestingly, in comparison to control neurons demyelinated axons showed substantially lower fidelity for input frequencies ≥400 Hz. The results raise the question, where APs in demyelinated axons fail? The underlying mechanisms and location of propagation failures may be complex. Increased failures in collaterals from demyelinated axons may be an integrated result of slower propagation and AP waveform changes. Indeed, the measured broad presynaptic AP predicts a longer duration to recover from Nav channel inactivation and delay the availability of inward current for the next spike. In addition, loss of voltage-gated Nav channels in demyelinated nodes preceding the presynaptic AP also will deteriorate AP regeneration at the BP and as a consequence limit invasion during high frequency spiking. In neocortical pyramidal neurons every AP during a high-frequency burst of ~250 Hz is initiated within the AIS (Kole, 2011). However, our results are not excluding the possibility that very high-frequency APs ≥400 Hz are actually initiated downstream the AIS, within the noR. Strong depolarization evoked at the soma electrotonically spreads down the axon and may increase the time to recover from Nav channel inactivation in the most proximal regions of the axon. A better detectability of high-frequency spikes has been described in distal axons of CA1 neurons during the complex spike (Apostolides et al., 2016). In this view, the failure of high-frequency presynaptic APs in demyelinated axons may be an *initiation-* rather then *propagation* failure. To identify the specific location where failure (or re-initiation) of the AP occurs during presynaptically recorded failures is technically challenging. Testing this hypothesis would require multiple loose-seal recordings from the axon proper, BPs and collaterals. These approaches are in particular difficult due to the extremely narrow stretch of exposed membrane accessible to the patch pipette tip in myelinated axons.

The observation of both structural and functional changes within the collaterals of demyelinated axons raises the question what the long-lasting functional consequences are for synaptic transmission. Dynamic reorganization of the presynaptic terminals is likely to impact on synaptic transmission and analysis of these sites may increase our understanding of the computational capacity of demyelinated neural circuits. Indeed, cuprizone-induced demyelination reduces the AMPA receptors in the hippocampus (Dutta et al., 2013). Furthermore, axon sprouting is consistent with the observed structural plasticity of axons within the cortico-spinal tract in the experimental autoimmune encephalomyelitis (EAE) model (Kerschensteiner et al., 2004). In the adult mammalian central nervous system myelin exerts an inhibitory influence on axon elongation and regeneration (Schwab and Bartholdi, 1996; Horner and Gage, 2000) mediated, among other players, by oligodendrocyte myelin-associated glycoprotein (MAG; McKerracher et al., 1994; Mukhopadhyay et al., 1994), and Nogo-A (Chen et al., 2000; GrandPré et al., 2000). These proteins are thought to limit axon growth and confine plasticity within restricted regions, preventing the formation of aberrant connections. Unlike EAE the cuprizone model is a toxicological model, which induces demyelination by selectively killing mature oligodendrocytes with minimal inflammatory responses (Kipp et al., 2009). It is possible that in both demyelination models the axons are no longer exposed to the growth inhibition mediated by myelin-associated proteins, possibly permitting structural plasticity along the previously myelinated internodes. Consistent with this idea previous studies have reported a significant decrease of Nogo-A levels in cuprizone-treated animals (Kuhlmann et al., 2007; Skripuletz et al., 2010).

Taken together, the present results show that oligodendrocyte loss in the gray matter causes a wide range of site-specific structural and functional changes throughout the axon arborization including its presynaptic boutons. While demyelinated axons show a loss of rapid impulse saltation they are also characterized by geometrical *de novo* branch outgrowth and reduced ability to propagate APs at high frequencies. Structural reorganization of the glutamatergic axonal collateral network may explain the aberrant intra-cortical excitation and hyperexcitability of demyelinated cortical neural circuits (Hamada and Kole, 2015).

## Author Contributions

MHPK: conceptualization, visualization, supervision, and funding acquisition; MSH, MAP and MHPK: methodology, analysis, review writing and editing; MSH and MAP: investigation; MSH and MHPK: writing the original draft.

## Funding

This work has been funded by a National Multiple Sclerosis Society Grant (RG 4924A1/1) and European Research Council Starting Grant (ERC StG 261114, EU 7th Framework) to MHPK.

## Conflict of Interest Statement

The authors declare that the research was conducted in the absence of any commercial or financial relationships that could be construed as a potential conflict of interest.

## References

[B1] Apostolides P. F. Milstein A. D. Grienberger C. Bittner K. C. Magee J. C. (2016). Axonal filtering allows reliable output during dendritic plateau-driven complex spiking in CA1 neurons. Neuron 89, 770–783. 10.1016/j.neuron.2015.12.040 26833135

[B2] Arroyo E. J. Xu T. Grinspan J. Lambert S. Levinson S. R. Brophy P. J.. (2002). Genetic dysmyelination alters the molecular architecture of the nodal region. J. Neurosci. 22, 1726–1737. 11880502 10.1523/JNEUROSCI.22-05-01726.2002 PMC6758867

[B3] Bagchi B. Al-Sabi A. Kaza S. Scholz D. O’Leary V. B. Dolly J. O.. (2014). Disruption of myelin leads to ectopic expression of K_V_1.1 channels with abnormal conductivity of optic nerve axons in a cuprizone-induced model of demyelination. PLoS One 9:e87736. 10.1371/journal.pone.0087736 24498366 PMC3912067

[B4] Black J. A. Waxman S. G. Smith K. J. (2006). Remyelination of dorsal column axons by endogenous Schwann cells restores the normal pattern of Nav1.6 and Kv1.2 at nodes of Ranvier. Brain 129, 1319–1329. 10.1093/brain/awl057 16537565

[B5] Bostock H. Sears T. A. (1978). The internodal axon membrane: electrical excitability and continuous conduction in segmental demyelination. J. Physiol. 280, 273–301. 10.1113/jphysiol.1978.sp012384 690876 PMC1282659

[B6] Brecht M. Schneider M. Sakmann B. Margrie T. W. (2004). Whisker movements evoked by stimulation of single pyramidal cells in rat motor cortex. Nature 427, 704–710. 10.1038/nature02266 14973477

[B7] Chang K.-J. Zollinger D. R. Susuki K. Sherman D. L. Makara M. A. Brophy P. J.. (2014). Glial ankyrins facilitate paranodal axoglial junction assembly. Nat. Neurosci. 17, 1673–1681. 10.1038/nn.3858 25362471 PMC4260775

[B8] Chen M. S. Huber A. B. van der Haar M. E. Frank M. Schnell L. Spillmann A. A.. (2000). Nogo-A is a myelin-associated neurite outgrowth inhibitor and an antigen for monoclonal antibody IN-1. Nature 403, 434–439. 10.1038/35000219 10667796

[B9] Clarner T. Diederichs F. Berger K. Denecke B. Gan L. van der Valk P.. (2012). Myelin debris regulates inflammatory responses in an experimental demyelination animal model and multiple sclerosis lesions. Glia 60, 1468–1480. 10.1002/glia.22367 22689449

[B10] Craner M. J. Newcombe J. Black J. A. Hartle C. Cuzner M. L. Waxman S. G. (2004). Molecular changes in neurons in multiple sclerosis: altered axonal expression of Nav1.2 and Nav1.6 sodium channels and Na^+^/Ca^2+^ exchanger. Proc. Natl. Acad. Sci. U S A 101, 8168–8173. 10.1073/pnas.0402765101 15148385 PMC419575

[B11] Crawford D. K. Mangiardi M. Xia X. López-Valdés H. E. Tiwari-Woodruff S. K. (2009). Functional recovery of callosal axons following demyelination: a critical window. Neuroscience 164, 1407–1421. 10.1016/j.neuroscience.2009.09.069 19800949

[B13] Debanne D. Guérineau N. C. Gähwiler B. H. Thompson S. M. (1997). Action-potential propagation gated by an axonal I(A)-like K^+^ conductance in hippocampus. Nature 389, 286–289. 10.1038/38502 9305843

[B14] Deschênes M. Landry P. (1980). Axonal branch diameter and spacing of nodes in the terminal arborization of identified thalamic and cortical neurons. Brain Res. 191, 538–544. 10.1016/0006-8993(80)91302-5 7378769

[B12] de Waegh S. M. Lee V. M. Brady S. T. (1992). Local modulation of neurofilament phosphorylation, axonal caliber, and slow axonal transport by myelinating Schwann cells. Cell 68, 451–463. 10.1016/0092-8674(92)90183-d 1371237

[B15] Ducreux C. Reynaud J. C. Puizillout J. J. (1993). Spike conduction properties of T-shaped C neurons in the rabbit nodose ganglion. Pflugers Arch. 424, 238–244. 10.1007/bf00384348 8414912

[B16] Dutta R. Chomyk A. M. Chang A. Ribaudo M. V. Deckard S. A. Doud M. K.. (2013). Hippocampal demyelination and memory dysfunction are associated with increased levels of the neuronal microRNA miR-124 and reduced AMPA receptors. Ann. Neurol. 73, 637–645. 10.1002/ana.23860 23595422 PMC3679350

[B17] Felts P. A. Baker T. A. Smith K. J. (1997). Conduction in segmentally demyelinated mammalian central axons. J. Neurosci. 17, 7267–7277. 9295373 10.1523/JNEUROSCI.17-19-07267.1997 PMC6573430

[B18] Foust A. J. Yu Y. Popovic M. A. Zecevic D. McCormick D. A. (2011). Somatic membrane potential and kv1 channels control spike repolarization in cortical axon collaterals and presynaptic boutons. J. Neurosci. 31, 15490–15498. 10.1523/JNEUROSCI.2752-11.2011 22031895 PMC3225031

[B19] Fraher J. P. Kaar G. F. (1984). The transitional node of Ranvier at the junction of the central and peripheral nervous systems: an ultrastructural study of its development and mature form. J. Anat. 139, 215–238. 6490515 PMC1164371

[B20] Goldstein S. S. Rall W. (1974). Changes of action potential shape and velocity for changing core conductor geometry. Biophys. J. 14, 731–757. 10.1016/s0006-3495(74)85947-3 4420585 PMC1334570

[B21] GrandPré T. Nakamura F. Vartanian T. Strittmatter S. M. (2000). Identification of the Nogo inhibitor of axon regeneration as a Reticulon protein. Nature 403, 439–444. 10.1038/35000226 10667797

[B22] Hamada M. S. Kole M. H. P. (2015). Myelin loss and axonal ion channel adaptations associated with gray matter neuronal hyperexcitability. J. Neurosci. 35, 7272–7286. 10.1523/JNEUROSCI.4747-14.2015 25948275 PMC4420788

[B23] Harris K. D. Mrsic-Flogel T. D. (2013). Cortical connectivity and sensory coding. Nature 503, 51–58. 10.1038/nature12654 24201278

[B24] Horner P. J. Gage F. H. (2000). Regenerating the damaged central nervous system. Nature 407, 963–970. 10.1038/35039559 11069169

[B25] Kerschensteiner M. Bareyre F. M. Buddeberg B. S. Merkler D. Stadelmann C. Brück W.. (2004). Remodeling of axonal connections contributes to recovery in an animal model of multiple sclerosis. J. Exp. Med. 200, 1027–1038. 10.1084/jem.20040452 15492125 PMC2211840

[B26] Khaliq Z. M. Raman I. M. (2006). Relative contributions of axonal and somatic Na channels to action potential initiation in cerebellar Purkinje neurons. J. Neurosci. 26, 1935–1944. 10.1523/JNEUROSCI.4664-05.2006 16481425 PMC6674931

[B27] Kim J. H. Renden R. von Gersdorff H. (2013). Dysmyelination of auditory afferent axons increases the jitter of action potential timing during high-frequency firing. J. Neurosci. 33, 9402–9407. 10.1523/JNEUROSCI.3389-12.2013 23719808 PMC3719047

[B28] Kipp M. Clarner T. Dang J. Copray S. Beyer C. (2009). The cuprizone animal model: new insights into an old story. Acta Neuropathol. 118, 723–736. 10.1007/s00401-009-0591-3 19763593

[B29] Kole M. H. P. (2011). First node of ranvier facilitates high-frequency burst encoding. Neuron 71, 671–682. 10.1016/j.neuron.2011.06.024 21867883

[B33] Kole M. H. P. Letzkus J. J. Stuart G. J. (2007). Axon initial segment Kv1 channels control axonal action potential waveform and synaptic efficacy. Neuron 55, 633–647. 10.1016/j.neuron.2007.07.031 17698015

[B30] Kole M. H. P. Popovic M. A. (2016). “Patch-clamp recording from myelinated central axons,” in Advanced Patch-Clamp Analysis for Neuroscientists, ed. Korngreen A. (New York, NY: Springer), 123–138.

[B31] Kole M. H. P. Stuart G. J. (2008). Is action potential threshold lowest in the axon? Nat. Neurosci. 11, 1253–1255. 10.1038/nn.2203 18836442

[B32] Kole M. H. P. Stuart G. J. (2012). Signal processing in the axon initial segment. Neuron 73, 235–247. 10.1016/j.neuron.2012.01.007 22284179

[B34] Kuhlmann T. Remington L. Maruschak B. Owens T. Brück W. (2007). Nogo-A is a reliable oligodendroglial marker in adult human and mouse CNS and in demyelinated lesions. J. Neuropathol. Exp. Neurol. 66, 238–246. 10.1097/01.jnen.0000248559.83573.71 17356385

[B35] Manor Y. Koch C. Segev I. (1991). Effect of geometrical irregularities on propagation delay in axonal trees. Biophys. J. 60, 1424–1437. 10.1016/s0006-3495(91)82179-8 1777567 PMC1260202

[B36] Mason J. L. Langaman C. Morell P. Suzuki K. Matsushima G. K. (2001). Episodic demyelination and subsequent remyelination within the murine central nervous system: changes in axonal calibre. Neuropathol. Appl. Neurobiol. 27, 50–58. 10.1046/j.0305-1846.2001.00301.x 11299002

[B37] McDonald W. I. Sears T. A. (1970). The effects of experimental demyelination on conduction in the central nervous system. Brain 93, 583–598. 10.1093/brain/93.3.583 4319185

[B38] McKerracher L. David S. Jackson D. L. Kottis V. Dunn R. J. (1994). Identification of myelin-associated glycoprotein as a major myelin-derived inhibitor of neurite growth. Neuron 13, 805–811. 10.1016/0896-6273(94)90247-x 7524558

[B39] Monsivais P. Clark B. A. Roth A. Häusser M. (2005). Determinants of action potential propagation in cerebellar Purkinje cell axons. J. Neurosci. 25, 464–472. 10.1523/JNEUROSCI.3871-04.2005 15647490 PMC6725482

[B40] Mukhopadhyay G. Doherty P. Walsh F. S. Crocker P. R. Filbin M. T. (1994). A novel role for myelin-associated glycoprotein as an inhibitor of axonal regeneration. Neuron 13, 757–767. 10.1016/0896-6273(94)90042-6 7522484

[B41] Parnas I. Segev I. (1979). A mathematical model for conduction of action potentials along bifurcating axons. J. Physiol. 295, 323–343. 10.1113/jphysiol.1979.sp012971 521942 PMC1279048

[B42] Poliak S. Peles E. (2003). The local differentiation of myelinated axons at nodes of Ranvier. Nat. Rev. Neurosci. 4, 968–980. 10.1038/nrn1253 14682359

[B43] Popovic M. A. Foust A. J. McCormick D. A. Zecevic D. (2011). The spatio-temporal characteristics of action potential initiation in layer 5 pyramidal neurons: a voltage imaging study. J. Physiol. 589, 4167–4187. 10.1113/jphysiol.2011.209015 21669974 PMC3180577

[B46] Rasband M. N. Peles E. Trimmer J. S. Levinson S. R. Lux S. E. Shrager P. (1999). Dependence of nodal sodium channel clustering on paranodal axoglial contact in the developing CNS. J. Neurosci. 19, 7516–7528. 10460258 10.1523/JNEUROSCI.19-17-07516.1999 PMC6782503

[B45] Rasband M. N. Shrager P. (2000). Ion channel sequestration in central nervous system axons. J. Physiol. 525, 63–73. 10.1111/j.1469-7793.2000.00063.x 10811725 PMC2269925

[B47] Rasband M. N. Trimmer J. S. Schwarz T. L. Levinson S. R. Ellisman M. H. Schachner M.. (1998). Potassium channel distribution, clustering and function in remyelinating rat axons. J. Neurosci. 18, 36–47. 9412484 10.1523/JNEUROSCI.18-01-00036.1998 PMC6793423

[B48] Romand S. Wang Y. Toledo-Rodriguez M. Markram H. (2011). Morphological development of thick-tufted layer v pyramidal cells in the rat somatosensory cortex. Front. Neuroanat. 5:5. 10.3389/fnana.2011.00005 21369363 PMC3043270

[B49] Rowan M. J. M. DelCanto G. Yu J. J. Kamasawa N. Christie J. M. (2016). Synapse-level determination of action potential duration by K^+^ channel clustering in axons. Neuron 91, 370–383. 10.1016/j.neuron.2016.05.035 27346528 PMC4969170

[B50] Rowan M. J. M. Tranquil E. Christie J. M. (2014). Distinct Kv channel subtypes contribute to differences in spike signaling properties in the axon initial segment and presynaptic boutons of cerebellar interneurons. J. Neurosci. 34, 6611–6623. 10.1523/JNEUROSCI.4208-13.2014 24806686 PMC4012316

[B51] Schwab M. E. Bartholdi D. (1996). Degeneration and regeneration of axons in the lesioned spinal cord. Physiol. Rev. 76, 319–370. 8618960 10.1152/physrev.1996.76.2.319

[B52] Shrager P. (1993). Axonal coding of action potentials in demyelinated nerve fibers. Brain Res. 619, 278–290. 10.1016/0006-8993(93)91622-y 8397054

[B53] Skripuletz T. Bussmann J.-H. Gudi V. Koutsoudaki P. N. Pul R. Moharregh-Khiabani D.. (2010). Cerebellar cortical demyelination in the murine cuprizone model. Brain Pathol. 20, 301–312. 10.1111/j.1750-3639.2009.00271.x 19371354 PMC8094790

[B54] Sloper J. J. Powell T. P. (1979). A study of the axon initial segment and proximal axon of neurons in the primate motor and somatic sensory cortices. Philos. Trans. R. Soc. Lond. B Biol. Sci. 285, 173–197. 10.1098/rstb.1979.0004 88058

[B55] Smith C. M. Cooksey E. Duncan I. D. (2013). Myelin loss does not lead to axonal degeneration in a long-lived model of chronic demyelination. J. Neurosci. 33, 2718–2727. 10.1523/JNEUROSCI.4627-12.2013 23392698 PMC4128634

